# Standardized evaluation protocols for deceased and living liver donors: a practical framework for emerging liver transplant programs

**DOI:** 10.3389/frtra.2026.1739776

**Published:** 2026-02-20

**Authors:** Pablo Coste Murillo, Rodrigo Álvarez Buitrago, Vanessa López Jara, María Fernanda Lynch-Mejía, Francisco Vargas Navarro, Wagner Ramírez Quesada, Christie Perelló, José Luis Calleja

**Affiliations:** 1 Liver Unit, Hospital R.A. Calderón Guardia, San José, Costa Rica; 2 LiverLab CR, San José, Costa Rica; 3 Department of Gastroenterology and Hepatology, Hospital Universitario Puerta de Hierro Majadahonda, IDIPHIM, Madrid, Spain; 4 Liver Unit, Center for Digestive Studies, Hospital Metropolitano de Santiago, Dominican Republic

**Keywords:** deceased donor, donor evaluation, emerging programs, extended criteria donor, liver transplantation, living donor, transplant protocol

## Abstract

**Background:**

Liver transplantation (LT) remains the definitive therapy for end-stage liver disease. However, significant variability in infrastructure, policy, and clinical practice continues to influence the implementation of deceased donor (DDLT) and living donor liver transplantation (LDLT) worldwide, particularly across emerging and expanding programs.

**Methods:**

This narrative review synthesizes contemporary guidelines, expert consensus documents, and high-impact clinical studies on donor evaluation. It presents a standardized and pragmatic framework for both DDLT and LDLT, integrating medical, radiologic, ethical, and psychosocial domains. Protocols are designed to be evidence-based, reproducible, and aligned with international standards.

**Key content and findings:**

In DDLT, optimal donor management, accurate neurological determination of death, and comprehensive infectious disease screening are essential for graft viability. In LDLT, meticulous psychosocial and anatomical assessments remain critical to donor safety. Advances such as machine perfusion, desensitization protocols, and expanded donor criteria have improved outcomes and broadened transplant opportunities. The proposed framework consolidates global best practices to support program consistency and quality assurance.

**Conclusions:**

This review provides a comprehensive and practical approach to donor evaluation in LT, promoting harmonization of practices across diverse healthcare systems. Its adoption may enhance donor safety, optimize graft utilization, and support the sustainable growth of both DDLT and LDLT programs worldwide.

## Graphical Abstract

**Figure:**
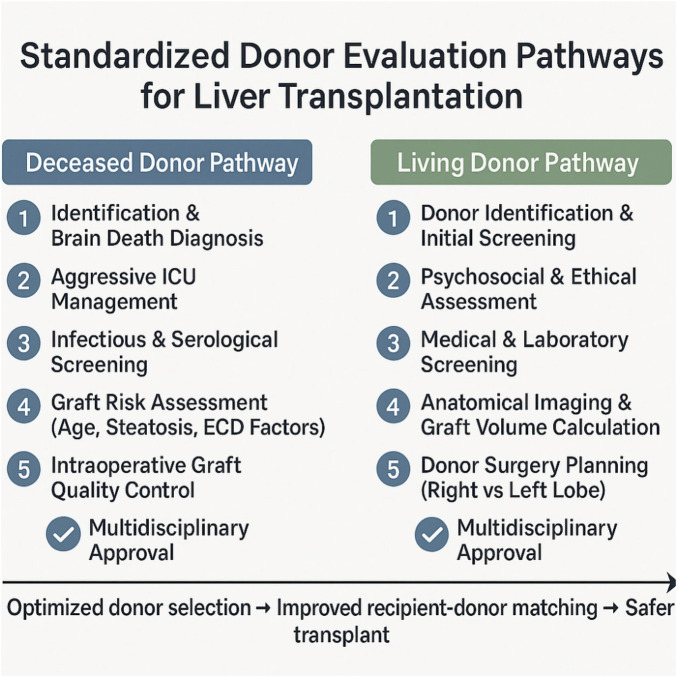

## Highlights

Practical framework for standardized donor evaluation in emerging and expanding liver transplant programs.
Integrates protocols for both deceased and living donor liver transplantation.
Multidisciplinary, evidence-based approach to enhance donor safety and evaluation consistency.
Supports responsible donor pool expansion while maintaining ethical and clinical safety standards.

## 1 Introduction

Despite the remarkable success of liver transplantation (LT) as a life-saving therapy, a widening gap persists between the number of patients in need and the availability of donor organs (1). This imbalance reflects multiple contributing factors, including the global rise in liver disease burden, limited donor registration and recovery rates, and logistical constraints in organ procurement and allocation systems (1–3).

Cultural and socioeconomic differences influence donor types across regions. In Asia, more than 90% of liver transplants are performed using living donors (LD), whereas in most Western countries a similar proportion relies on deceased donors (DD) (4). Consequently, living donor liver transplantation (LDLT) remains markedly underutilized across the Western world, representing only 5%–10% of all liver transplants in the United States (3). A comparable trend is observed in Latin America, where a 2019 survey of 143 transplant centers across 15 countries found that only 12.5% of the 3,837 transplants performed involved living donors, underscoring the limited adoption of LDLT in the region (5).

**Figure 1:**
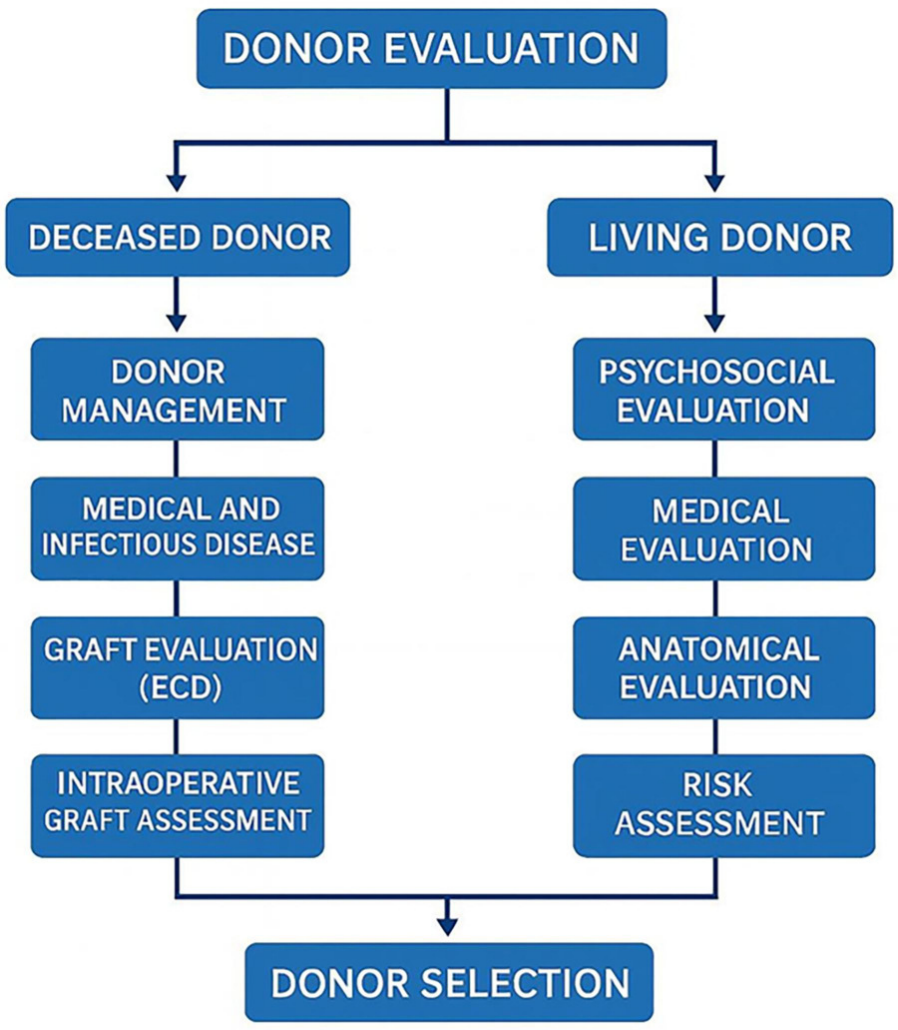
Evaluation algorithm summarizing the key steps for DDLT/LDLT programs. This flowchart outlines the structured evaluation process for both deceased and living liver donors. For deceased donors, the assessment includes donor management, medical and infectious disease screening, graft evaluation—including consideration of extended criteria donors (ECD)—and intraoperative graft assessment. For living donors, the pathway begins with psychosocial evaluation, followed by detailed medical and anatomical assessment, and concludes with a comprehensive risk-benefit analysis to ensure donor safety and transplant feasibility. Both processes culminate in multidisciplinary donor selection.

While DDLT is generally considered technically less complex and offers larger grafts, donor quality and donation rates often remain suboptimal in many low- and middle-income countries (5, 6). Conversely, LDLT offers several distinct advantages: it reduces waiting time, confers a clear survival advantage over remaining on the waitlist, with significant life-years gained, and improves overall access to transplantation by expanding the donor pool (3, 7). Moreover, LDLT enables elective surgical planning for urgent or high-risk candidates, ensures superior graft quality through donor optimization, and facilitates transplantation in cases with extended indications such as hepatocellular carcinoma, cholangiocarcinoma, metastatic liver disease, or low MELD scores. However, LDLT also introduces unique ethical, medical, and surgical challenges and entails inherent risks for the donor (8, 9) (Figure 1).

In both modalities, rigorous donor evaluation is essential to ensure safety, optimize graft outcomes, and uphold ethical transplantation practices. This review summarizes current practices in DD and LD evaluation and proposes a practical, evidence-based framework to guide emerging and developing liver transplant programs, providing an overview of updated international standards and best practices that can be adapted across diverse healthcare settings.

## 2 Deceased donor evaluation (DDLT)

### 2.1 Medical evaluation and pre-operative optimization

Optimal recipient outcomes in DDLT depend on comprehensive donor evaluation and meticulous intensive care unit (ICU) management aimed at preserving organ function. Donor evaluation should include comprehensive medical and behavioral history, physical examination, and laboratory workup. Core tests encompass ABO blood typing, complete blood count (CBC), electrolytes, renal and hepatic function tests, coagulation profile, glucose, and a comprehensive infectious screening panel (HBsAg, anti-HBc, anti-HBs, anti-HCV, anti-HIV, CMV, EBV, VDRL, toxoplasmosis, varicella-zoster virus, HTLV-I/II, HSV-I/II, *Trypanosoma cruzi*, and SARS-CoV-2).

Depending on the donor's origin and local epidemiology, expanded screening for endemic pathogens, such as *Strongyloides stercoralis, West Nile virus*, malaria, and Leptospira species, may be warranted. Additional evaluations include arterial blood gases, urinalysis, chest radiography, abdominal ultrasound, and, when hospitalization exceeds 72 h, blood, and urine cultures (10–12). Pre-procurement liver biopsy (LB) and computed tomography (CT) Imaging are recommended when steatosis, focal lesions, or vascular abnormalities are suspected.

Most DDLT grafts originate from donors after brain dead (DBD). Brain death is defined as the complete and irreversible cessation of all brain activity, including the brainstem (13). Diagnosis is based primarily on clinical criteria and bedside neurological examination, while ancillary testing is reserved for cases with diagnostic uncertainty (14). Legal and ethical requirements for death certification and consent vary by jurisdiction and must be strictly observed before any procurement procedure (13, 14).

Goal-directed donor management is essential to maintain hemodynamic and metabolic homeostasis, thereby preserving graft quality (15, 16). Recommended targets include: mean arterial pressure >60 mmHg, urine output >1 ml/kg/h, temperature >35 °C, serum sodium <150 mEq/L, lactate normalization, PaO2 > 80 mmHg, hemoglobin >7 g/dl, hematocrit >30%, glucose <180 mg/dl, central venous pressure <10 mmHg, pH 7.35–7.45, caloric intake 70%–85% of baseline needs, fibrinogen >100 mg/dl, platelets >80,000/mm^3^, and hormonal support (e.g., thyroid and corticosteroids) (17–21).

In many regions, effective donor evaluation relies on Organ Procurement Organizations (OPOs) or equivalent procurement networks that operate independently from the recipient team. This organizational separation ensures transparency, minimizes conflicts of interest, and allows specialized personnel to focus exclusively on donor optimization (22).

Beyond DBD, donation after circulatory death (DCD) has become an increasingly relevant source of grafts, particularly with the advent of machine perfusion technologies. Although DCD donation presents unique challenges related to warm ischemia and early graft dysfunction, advances in hypothermic and normothermic perfusion have markedly improved outcomes, enabling the safe expansion of this donor pool (23, 24).

### 2.2 Graft function evaluation

Given the shortage of organs, the use of marginal or extended criteria donors (ECDs) has increased. Although there is no universally accepted definition of ECDs, they are generally associated with higher risks of early allograft dysfunction (EAD), suboptimal graft function, or transmission of donor-derived diseases (25). Emerging and developing liver transplant programs, apply varying acceptance criteria depending on local policies, access to LDLT, and the risk of patient mortality (5, 6, 25).

Key donor risk factors include:

Age: No strict upper limit exists, but donors >60 years require meticulous evaluation (25–29).
Cold ischemia time <8 h may optimize outcomes in elderly donors (11, 30–32). Functional warm ischemia (SBP <50 mmHg or SpO₂ < 80% to cold flush) should not exceed 30 min in standard DCD procurement (33, 34).
Steatosis: Mild steatosis (<30%) has minimal impact on graft outcomes. In contrast, moderate to severe macrovesicular steatosis is associated with an increased risk of severe preservation injury, primary non-function, delayed graft function, and graft failure (25, 26, 35, 36). When uncertainty exists, LB remains the gold standard for evaluation (37, 38). Microvesicular steatosis, even when moderate, is not associated with worse outcomes (39).
Liver Enzymes: In transplanted livers, several studies have shown that markers of ischemic liver injury and recovery, such as peak ALT levels and the change from peak ALT to terminal ALT prior to transplant, are not associated with graft outcomes. Therefore, no definitive upper cut-off for graft acceptance based on these parameters has been established (40, 41). However, a marked increase in GGT (over 200 UI/L) should be carefully evaluated, and according to some consensus guidelines, a liver biopsy is recommended in such cases (25, 26).
Hypernatremia: Early reports associated donor hypernatremia with increased rates of graft loss, poor initial graft function, and higher post-transplant mortality (42–44). Specifically, a serum sodium level >155 mEq/L was linked to worse outcomes within the first month after LT (45, 46). However, subsequent studies have failed to confirm a consistent association with poor prognosis. To date, donor serum sodium levels alone do not appear to have a clinically significant impact on post-transplant liver function (47–52).
Sepsis/infection: A major UNOS study found that donors with positive blood cultures were associated with reduced graft survival, though patient survival was unaffected (53). Conversely, more recent studies did not report adverse outcomes when using bacteremic DD (54–56). Donor infection is not an absolute contraindication for liver transplantation. However, grafts from potentially septic donors should meet the following criteria: non-hepatic infection source, effective antibiotic coverage for at least 24–48 h, and absence of multidrug-resistant organisms (11).
Hepatitis C and B viruses (HCV, HBV): In the post–direct-acting antiviral (DAA) era, the use of HCV-viremic donors has shown excellent outcomes in both HCV-positive and HCV-negative recipients (57–59). Similarly, liver transplantation from hepatitis B core antibody–positive (HBcAb+) donors, when combined with appropriate prophylaxis [nucleos(t)ide analogues and hepatitis B immunoglobulin], yields outcomes comparable to HBcAb-negative grafts (60–62). As a result, these donors are increasingly accepted in clinical practice. Recent series demonstrate that carefully selected HBsAg-positive donor grafts transplanted into HBV-negative recipients can achieve survival exceeding 90% at 3 years when the donor liver is non-fibrotic and HBV DNA is undetectable (63). Patients must be carefully counseled about the risks and benefits.
Malignancy: Although rare, donor-transmitted cancers (DTCs) represent a low but nonzero risk (12, 64). Graft acceptance should be individualized following thorough clinical, laboratory, and radiologic evaluation, and discussed by a multidisciplinary team considering cancer type, stage, disease-free interval, and transplant urgency. Recent guidelines recommend additional imaging (e.g., CT) in high-risk donors, exclusion of malignancy or intracranial metastases in cases of intracranial bleeding, and Beta-human chorionic gonadotropin (β-HCG) testing in women of childbearing age with unexplained intracranial hemorrhage (12). Several resources are available to guide decision-making in specific scenarios (12, 64–66).

Several scoring systems have been developed to assess donor risk based on clinical parameters, with the Donor Risk Index (DRI) being the most widely used (67–72). However, its applicability is limited in the current era of refined surgical techniques, enhanced perioperative management, and machine perfusion, highlighting the need for recalibration (73). Emerging tools—such as artificial intelligence, molecular profiling (multi-omics, single-cell technologies), and machine perfusion—offer promising avenues for improving graft evaluation (74–79).

Advances in dynamic preservation have reshaped the evaluation of ECD and DCD grafts. Hypothermic oxygenated perfusion (HOPE) reduces ischemia–reperfusion injury by restoring mitochondrial respiration and promoting metabolic recovery prior to implantation, consistently lowering rates of early allograft dysfunction (80). Normothermic machine perfusion (NMP), by maintaining physiological temperature, allows real-time functional assessment through lactate clearance, glucose metabolism, bile production, and bile quality (pH or bicarbonate content), and perfusate transaminase trends (24, 81). Both modalities have demonstrated improved outcomes in DCD grafts and enable prolonged preservation beyond traditional cold-storage limits, facilitating broader graft utilization while supporting objective viability assessment (24, 80).

At the time of organ retrieval, there is no standardized criterion for determining when a LB is necessary; this decision remains center- and surgeon-dependent (82, 83). The pathologist plays a key role in interpreting histological findings within the broader donor risk context (84, 85).

Historically, the concept of donor–recipient risk matching has guided allocation strategies in LT. Although no randomized controlled trial has definitively established a rigid algorithm, recent evidence supports aligning graft quality with recipient condition, that is, allocating higher-risk or ECD livers to physiologically robust, typically lower-MELD recipients, while reserving optimal grafts for those with higher urgency or comorbid burden. This risk-balancing approach aims to maximize graft utility and overall survival across the waiting list (86).

Ultimately, graft acceptance should be determined by a multidisciplinary team, balancing graft quality, recipient urgency, and center experience. Informed consent should include disclosure of any allograft-specific risks (25).

Although volumetric considerations are traditionally emphasized in LDLT, graft- recipient size mismatch also represents an important risk factor in DDLT. Oversized grafts may be difficult to implant in smaller recipients and have been associated with impaired venous outflow, increased intra-abdominal pressure, and abdominal compartment syndrome, particularly in pediatric or sarcopenic adults (87). Pre-procurement CT volumetry and anthropometric assessment can assist in anticipating mismatch, while intraoperative measures, such as temporary abdominal closure or delayed fascial closure, may mitigate mechanical constraints. Incorporating basic size-matching principles into DDLT evaluation adds an additional layer of safety and may improve implantation outcomes in selected recipients (Table 1).

**Table 1:** Donor evaluation checklist for deceased donor liver transplantation (DDLT).

Step	Key components
1. Donor identification and intensive care management	• Identification and documentation: declaration of brain death and informed consent. • Intensive care management by goals: - Mean arterial pressure >60 mmHg - Urine output >1 mL/kg/h - Temperature >35 °C - Serum sodium <150 mEq/dl - Normal arterial blood lactate - PaO2 > 80 mmHg - Hemoglobin >7 g/dL and hematocrit >30% - Glucose levels <180 mg/dL - Central venous pressure <10 mmHg - pH between 7.35–7.45 - Caloric intake: 70%–85% of baseline energy expenditure - Fibrinogen >100 mg/dl - Platelet count >80,000/mm^3^ - Thyroid and steroid replacement, among others.
2. Medical evaluation	a) Complete medical/behavioral history and physical examination: - Includes blood pressure, vasopressor doses, urine output, temperature, ventilation parameters. b) Laboratory tests: - Blood type, hematology tests, metabolic panel, liver function tests (LFTs), glucose - Basic serological testing: HBsAg, total anti-HBc, anti-HCV, anti-HIV, anti-CMV, anti-EBV, VDRL, toxoplasma IgG, VZV, HTLV-I/II, HSV-I/II, Chagas disease, SARS-CoV-2 - Coagulation tests, arterial blood gases, urinalysis c) Radiology: - Chest x-rays, abdominal ultrasonography d) Other: - Blood and urine cultures if hospitalization >72 h - Pre-procurement liver biopsy (LB) and CT imaging may be required.
3. Graft function evaluation	• Evaluation of extended criteria donors (ECD), including: - Donor age, hepatic steatosis, abnormal LFTs, hypernatremia, sepsis, malignancy, etc. • Application of donor risk scores (e.g., DRI) when applicable • Multidisciplinary analysis and decision-making process
4. Intraoperative graft assessment	• Visual assessment of graft during retrieval • Liver biopsy (LB) may be required based on appearance or risk profile • Histological assessment by pathologist • Final decision by multidisciplinary transplant team • Recipient informed consent for graft-specific risks

This table summarizes the essential steps in the evaluation of deceased donors for liver transplantation, including clinical stabilization, comprehensive laboratory, and imaging work-up, assessment of extended criteria donor (ECD) risk factors, and intraoperative graft evaluation. MAP, mean arterial pressure; CVP, central venous pressure; LFTs, liver function tests; ABG, arterial blood gases; LB, liver biopsy; CT, computed tomography; US, ultrasound; DRI, donor risk index.

## 3 LDLT donor evaluation

### 3.1 Initial considerations

The first step in LDLT is identifying a suitable donor, with donor safety as the overriding priority (88, 89). A multidisciplinary transplant team should perform a comprehensive assessment encompassing ethical, psychosocial, medical, and anatomical evaluations (vascular and biliary integrity) (89, 90). The donor team should be separate and distinct from the recipient team to minimize any potential conflicts of interest or bias. Institutional protocols vary depending on local resources and experience, and only about 40% of potential donors ultimately qualify for donation (91) (Table 2).

**Table 2:** Donor evaluation process for living donor liver transplantation (LDLT).

Step	Key components
1. Psychosocial evaluation	a) Education about procedure and associated risks. b) Assessment of motivation and ability to provide informed consent.
2. Medical evaluation	c) Initial evaluation: age, ABO blood type, BMI. d) Full medical history and physical examination. e) Laboratory testing: - Hematologic and metabolic panels, TSH, LFTs - Serologies: HBsAg, anti-HBc, anti-HCV, anti-HIV, anti-CMV, anti-EBV, VDRL, toxoplasmosis IgG, VZV, HTLV-I/II, HSV-I/II, Chagas - Coagulation and hypercoagulability screening - Genetic screening (if blood relative with liver disease) - Pregnancy test (women <50 years) f) Imaging: chest x-ray, abdominal ultrasound, transient elastography g) Electrocardiogram (EKG)
3. Graft anatomy and function evaluation	h) Cross-sectional imaging (MRI/CT), including 3D volumetry and anatomical assessment of vasculature and biliary structures
4. Preoperative risk and further evaluation	i) Cardiopulmonary evaluation as indicated: echocardiogram, stress echocardiography, cardiac CT angiogram, coronary angiography, pulmonary function tests j) Autoimmune markers (ANA, AMA), infectious disease screening (TB, endemic pathogens) k) Consultations: social work, nutrition, transplant coordination, psychiatry, anesthesiology l) Selective liver biopsy (LB): unexplained abnormal LFTs, suspected metabolic disorders, steatosis ≥10% on imaging, or lack of advanced imaging

This table summarizes the multidisciplinary evaluation of potential living liver donors, including psychosocial readiness, comprehensive medical and radiologic assessment, anatomic suitability of the graft, and risk-based preoperative workup. Key abbreviations: LFTs, liver function tests; TSH, thyroid-stimulating hormone; MRI, magnetic resonance imaging; CT, computed tomography; EKG, electrocardiogram; LB, liver biopsy; ANA, anti-nuclear antibody; AMA, anti-mitochondrial antibody.

### 3.2 Psychosocial evaluation

Living donor liver transplantation (LDLT) represents a unique scenario in which the donor undergoes major surgery for purely altruistic reasons without any direct medical benefit. The foundational ethical principles of autonomy, beneficence, non-maleficence, and justice must be upheld, and the donor's risk must be carefully balanced against the recipient's potential benefit under the concept of clinical equipoise (88, 92, 93).

The Vancouver Forum established key tenets to optimize donor safety (94). It specifies that: a) the risk to the donor must be justified by a predictable and acceptable outcome in the recipient; b) graft and patient survival should be comparable to outcomes achieved with DDLT; and c) LDLT should provide a clear advantage over remaining on the DDLT waiting list.

Living-donor surgery carries an overall complication rate of approximately 10%–40% and a mortality rate of <1% (95–97). LDLT also entails a significant learning curve, with most programs achieving procedural consistency after 15–20 consecutive donor hepatectomies (90). Consequently, informed consent is paramount: donors must clearly understand potential risks, and center-specific outcome and complication data should be transparently disclosed.

A licensed mental-health professional should assess each donor's psychological readiness and document full informed consent (9, 89, 90, 98). Donors retain the absolute right to refuse or withdraw consent at any time before surgery (93, 99).

The International Liver Transplantation Society (ILTS) and the International LDLT Group recently convened the ILTS-iLDLT Consensus Conference on Living Donor Safety. Although its formal publication is pending, this global initiative provides updated recommendations on ethical, psychosocial, and procedural safeguards, further strengthening donor protection within LDLT programs.

### 3.3 Medical evaluation

A thorough clinical history, physical examination, and initial laboratory testing are essential to exclude contraindications. Common baseline studies include hematologic, biochemical, and serologic panels for viral hepatitis (HBsAg, anti-HBc, anti-HCV, anti-HIV, CMV, EBV), chronic liver disease (ANA, ferritin/iron saturation, ceruloplasmin, α1-antitrypsin, IgG), and infectious disease screening adapted to geographic endemicity (VDRL, toxoplasmosis, varicella-zoster virus, HTLV-I/II, HSV-I/II, Chagas disease, tuberculosis screening with QuantiFERON or PPD), as well as pregnancy testing in women under 50 years (9, 11, 100, 101).

Although not universally standardized, screening for hypercoagulable states (factor V Leiden, prothrombin G20210A mutation, antithrombin III, protein C and S deficiency, antiphospholipid antibodies, homocysteine) is advisable (9, 90, 102, 103). When cost is a concern, testing can be performed in phases, starting with basic serologies and expanding as indicated. There should be a low threshold for screening for genetic liver diseases, especially among related donor-recipient pairs (9, 104, 105).

If initial testing is acceptable, further evaluation guided by center-specific protocols may include chest radiography, electrocardiography, abdominal imaging (ideally CT and MRI/MRCP), cardiovascular assessment (echocardiography, stress testing, or CT angiography in selected cases), pulmonary function testing (in smokers, asthmatics, or donors >40 years), and age- or risk-adapted cancer screening (11, 12, 90).

Multidisciplinary assessment by hepatology, surgery, anesthesiology, psychiatry, social work, nutrition, and transplant coordination teams is also standard (11, 12, 90). LB is reserved for selected cases, such as unexplained abnormal liver function tests, suspected metabolic disease (e.g., Wilson's disease, α1-antitrypsin deficiency), or when imaging suggests significant steatosis and advanced noninvasive modalities are unavailable (106–108).

While donor age limits vary by region and center, most programs accept candidates between between 18 and 60 years (7, 28, 109). Although younger donors typically offer more favorable vascular characteristics—reduced atherosclerosis, less vascular tortuosity, and larger, more uniform vessel calibers—age alone is not an absolute contraindication (110–112). Outcomes among donors over 55–60 years are variable and associated with reduced regenerative capacity and higher complication rates; therefore, more rigorous evaluation is warranted in this group (28, 97). Donation beyond age 60 should generally be limited to high-volume centers with extensive LDLT experience.

Ideal living donors should have no significant comorbidities, or only well-controlled mild conditions, and no evidence of major end-organ dysfunction, including cardiopulmonary disease or active malignancy. For donors with metabolic risk factors (prediabetes, hypertension, hyperlipidemia, obesity), metabolic syndrome should be optimized, and further evaluation should include screening for metabolic dysfunction–associated steatohepatitis (MASH), fibrosis, and cardiac risk assessment (89).

Body mass index (BMI) thresholds vary internationally, ranging from >25 kg/m^2^ (Indian guidelines) to >40 kg/m^2^ (absolute contraindication in some U.S. centers), but there is no universally accepted cutoff (109, 113). Most Western and Latin American programs set an upper limit of 30–32 kg/m^2^, with select acceptance up to 35 kg/m^2^ only in isolated obesity without metabolic abnormalities and in centers with extensive LDLT experience (7, 9). Donors with BMI >30 kg/m^2^ should undergo non-invasive quantification of hepatic steatosis [MRI-Proton Density Fat Fraction (PDFF) or CAP] and, when uncertainty persists, LB to exclude steatohepatitis or fibrosis (9, 89, 100, 114, 115).

ABO compatibility remains the standard criterion; however, emerging desensitization protocols (rituximab, plasmapheresis, intravenous immunoglobulin) have yielded acceptable outcomes in carefully selected ABO-incompatible cases (116–123). Final decisions depend on institutional expertise, available resources, and local experience.

### 3.4 Anatomical and functional assessment

Once donor physical and psychological suitability is confirmed, a comprehensive evaluation of graft anatomy, size, and function is essential. This includes assessment of liver volume, vascular and biliary anatomy, and hepatic parenchymal quality (e.g., exclusion of fibrosis, significant steatosis, or liver injury), using imaging modalities such as CT, MRI/MRCP, angiography, cholangiography, transient elastography, and, when indicated, LB (89, 124).

Expert consensus recommends MRI/MRCP and CT angiography as preferred modalities for biliary and vascular mapping, respectively, with adaptation to local expertise and resource availability (108). For graft selection (right lobe, left lobe, left/caudate lobe, or dual grafts), 3D reconstructions are advised to optimize volumetric planning and detect anatomical variations that may influence surgical strategy (9).

MRI, ideally incorporating PDFF, provides superior sensitivity and negative predictive value for detecting low-grade steatosis and can often reduce the need for biopsy (108). LB may be considered in high-risk candidates, those with metabolic syndrome, suspected MASH, elevated liver enzymes, or uncertainty in MRI-PDFF accuracy.

Although no universal threshold for acceptable macrovesicular steatosis exists, most centers adopt a <10% cutoff on LB (90, 101). Importantly, MRI-PDFF and histologic steatosis do not correlate linearly; in the study by Qadri et al., PDFF and biopsy estimates were comparable at 5%, however, PDFF values beyond this level consistently underestimated histologic steatosis with up to a 3.5-fold difference (125) Hence, if aiming for <10% macrosteatosis on biopsy, these intermodality differences must be considered. High-volume Asian centers may accept 10%–30% steatosis in young, otherwise ideal donors (9, 126).

The extent of hepatectomy is guided by recipient volume requirements, donor remnant liver volume (RLV), and anatomic feasibility. The right lobe (≈60% of total liver volume) is most used for adult LDLT, followed by the left lobe (≈40%), while left lateral segment grafts (≈20%) are reserved for pediatric recipients. Adequate graft size is critical to avoid small-for-size syndrome (SFSS). Graft adequacy is determined using the graft-to-recipient weight ratio (GRWR) and the graft volume-to-standard liver volume ratio (GV/SLV). A GRWR ≥0.8% or GV/SLV ≥40% is generally recommended, though high-risk recipients may require higher targets (1.0%–1.2% or >45%) (127).

SFSS manifests as early post-transplant dysfunction with hyperbilirubinemia, coagulopathy, ascites, and/or encephalopathy in the absence of technical or vascular causes (90). It is associated with graft hyperperfusion, portal hypertension, and inadequate venous outflow (90, 128). Portal inflow modulation (PIM), through splenic embolization, ligation, splenectomy, or shunting, can mitigate this risk. With careful PIM, successful LDLT using smaller grafts (GRWR ≥0.6% or GV/SLV ≥25%) has been reported in high-volume Asian centers when donor quality is excellent and urgency is high (129–131). Each case should be evaluated individually, particularly when flow-modulation strategies are applied (132, 133).

Donor safety requires maintaining a residual liver volume (RLV/TLV) ≥30%, although factors such as donor age, sex, and steatosis may influence this threshold (101, 108). Selected low-risk donors (<50 years, minimal steatosis, preserved middle hepatic vein) may tolerate slightly lower remnants, but these should be exceptions in highly experienced centers with advanced intraoperative monitoring (134–136). Most programs adopt a strict ≥30% cutoff. As graft size increases, recipient benefit rises but donor risk escalates proportionally; hence, the global trend favors greater utilization of left lobe grafts to enhance donor safety (90, 128, 137–140). However, left lobe graft should be avoided in recipients with BMI >30, MELD >20, age >45 years, or severe portal hypertension (128, 141–144).

When a single partial graft is insufficient, dual-graft LDLT (e.g., two left lobes or a left and right lobe) may be an option in select high-volume centers. Though effective, this technique involves substantial technical and logistical complexity and should be limited to institutions with extensive experience (145, 146).

### 3.5 Expansion of indications

In transplant oncology, LDLT plays an increasingly pivotal role—particularly for patients undergoing downstaging or bridging therapies for hepatocellular carcinoma (HCC), or for those with limited access to deceased donor grafts, such as cases of colorectal liver metastases (CRLM). The elective and planned nature of LDLT allows time-sensitive coordination crucial for patients requiring documented tumor control before transplantation.

Given its voluntary nature, some centers cautiously extend LDLT indications beyond those of DDLT, including selected cases of cholangiocarcinoma, CRLM, or HCC beyond conventional criteria (Milan, UCSF), when favorable tumor biology (low AFP, PET-negativity, sustained response to therapy) suggests a meaningful survival benefit (147, 148).

Several high-volume Asian centers have reported positive outcomes in high-urgency settings such as acute liver failure or acute-on-chronic liver failure patients. Although consensus is lacking, decisions should be guided by multidisciplinary risk-benefit analysis and supported by expedited donor-recipient evaluation protocols (149–155). Success depends on appropriate risk matching between donor and recipient and robust perioperative planning.

In summary, LDLT offers a life-saving alternative where deceased donor organs are scarce. Nonetheless, donor selection criteria should remain conservative, particularly in developing or mid-volume centers. Thresholds for age, BMI, steatosis, and remnant liver volume must prioritize donor safety rather than expansion. Any deviation from these standards should occur only in experienced, high-volume programs with multidisciplinary expertise and structured mentorship.

## 4 Conclusion

LT remains the definitive therapy for end-stage liver disease; however, significant disparities persist in donor utilization and access, particularly in emerging programs. A standardized, multidisciplinary evaluation framework is essential to safeguard donor welfare, optimize graft function, and ensure equitable access to transplantation.

This review provides a comprehensive and pragmatic framework for donor evaluation in both deceased and living liver transplantation, aligned with current international guidelines. Beyond technical standards, there should be an emphasizes in the importance of peer mentorship, capacity building, and collaborative networks between established and developing programs. The implementation of standardized, stepwise protocols, combined with inter-institutional collaboration, remains fundamental to ensuring donor safety, maximizing graft utilization, and advancing equitable access to LT worldwide.
